# Room Acoustical Parameters as Predictors of Acoustic Comfort in Outdoor Spaces of Housing Complexes

**DOI:** 10.3389/fpsyg.2020.00344

**Published:** 2020-03-04

**Authors:** Armin Taghipour, Sahand Athari, Arnthrudur Gisladottir, Tessa Sievers, Kurt Eggenschwiler

**Affiliations:** ^1^Laboratory for Acoustics/Noise Control, Empa, Swiss Federal Laboratories for Materials Science and Technology, Dübendorf, Switzerland; ^2^Department of Engineering, Aarhus University, Aarhus, Denmark; ^3^The Carl von Ossietzky University of Oldenburg, Oldenburg, Germany

**Keywords:** acoustic comfort, inner yard, room acoustical parameters, psychoacoustic experiment, virtual acoustics

## Abstract

Room acoustical parameters have frequently been used to evaluate or predict the acoustical performance in rooms. For housing complexes in urban areas with high population density, it is important to improve acoustic performance not solely indoors, but outdoors as well; for example on the balconies or in the yards. This paper investigates to what extent classic room acoustical parameters would be able to predict the perceived acoustic comfort in outdoor spaces (i.e., courtyards) of virtual housing complexes. Individual and combined effects of a series of independent variables (such as facade absorption, sound source, and observer position) on short-term acoustic comfort were investigated in three laboratory experiments. ODEON software was used for virtual inner yard simulation, whereby 2D spatialization was carried out for a playback over five loudspeakers. Moderate facade absorption was found to increase acoustic comfort. Relatively pleasant and relatively unpleasant sounds were associated with comfort and discomfort, respectively. Lower acoustic comfort ratings were observed at receiver positions with high sound pressure levels and/or strong flutter echoes. A further analysis of the results is carried out here with respect to the room acoustical parameters and their ability to predict the acoustic comfort ratings. Speech transmission index (STI), definition (D50), clarity of speech (C50) and music (C80), early decay time (EDT), and lateral energy fraction (LF80) were found to be significantly correlated with acoustic comfort. They were found to be significant predictors of acoustic comfort in a series of linear mixed-effect models. Furthermore, linear mixed-effect models were established with the A-weighted equivalent continuous sound level, LAeq, as a significant predictor of acoustic comfort.

## 1. Introduction

Development and densification of urban areas has led to an alteration of the urban sound environment and many inhabitants are exposed to high noise levels in their everyday life. Noise emitted from classic noise sources (aircraft, railway etc.) has been related to several health implications and disturbances (WHO, 2018) and thus, the reduction of noise emission in urban areas has been the main objective of conventional and construction acoustics.

One approach to the mitigation of noise in urban living areas is the construction of housing complexes with courtyards or inner yards, where the buildings perform as shields, lowering sound levels from road traffic on one side of the buildings. This allows for several rooms of dwellings to face a “quiet” side of the building complex (Öhrström et al., 2006). Another advantage is that inner yards give access to a recreational outdoor space with lower sound levels from road traffic, which has the opportunity to support various needs of the residents for relaxation, sports or other activities (Gidlöf-Gunnarsson and Öhrström, 2010). Thus, inner yards of housing complexes are under investigation in Switzerland as building typologies with capacities for improvement of the sound environment and acoustic comfort (Sturm and Bürgin, 2016; Sievers et al., 2018; Sturm et al., 2019).

Gidlöf-Gunnarsson and Öhrström (2010) highlighted physical environmental aspects presence of which is found to be highly valuable within inner yards, one of them being the protection from disturbing noise. Although the housing complex benefits from a shielding effect from the street, the inhabitants are confronted with daily life sounds from within the yard itself (Taghipour et al., 2019b,c). Depending on material properties and the building structure among others, the housing complex can induce complicating acoustic effects within the yard, such as multiple reflections, diffraction, and diffusion (Yang et al., 2013). A sound pressure level (SPL) increase of up to 8 dB has been reported due to multiple reflections outside of an apartment complex in comparison to a semi-free field (Yang et al., 2013). Thus, an improvement of acoustic comfort in the building design could benefit the residents. As an example, the use of material with absorptive properties on surfaces outside housing complexes could reduce the SPL, which could then result in increased acoustic comfort (Calleri et al., 2017).

Although the use of sound absorbing materials is well known for the improvement of acoustic comfort in closed rooms/buildings (Battaglia, 2014; Xiao and Aletta, 2016; Thomas et al., 2018), less is known about the use of such materials for the improvement of acoustic comfort for residents of housing complexes with shared inner yards (Taghipour et al., 2019c). Within other exterior spaces of the urban layout, facade absorption has been found to affect the acoustic performance. In public squares, facade absorption has proved to be influential in the subjective assessment of space wideness (Calleri et al., 2017). Alongside streets, building facade and balcony absorption has been found to reduce levels from traffic noise (Lee et al., 2007; Yeung, 2016) and leisure noise (Badino et al., 2019) along facades. Hornikx and Forssén (2009) have found that the use of absorptive facade materials in a shielded canyon could lead to SPL reduction for various observer positions within the canyon. By combining the use of facade absorption and geometrical modification, such as in balcony design, building facades seem to be potentially effective mitigators of noise (Lee et al., 2007). In a study of the effect of facade shape and acoustic cladding on the reduction of leisure noise levels in a street canyon, Badino et al. (2019) have stated that by adding sound absorbing materials on a geometrically optimized facade, a reduction of up to 10 dB in the A-weighted SPL can be achieved. This includes optimized design of balconies, which can greatly influence the facade noise levels (Echevarria Sanchez et al., 2016; Badino et al., 2019). Generally, balconies on building facades have been found to provide significant protection from a noise source on the ground or on the roadway (Hossam El Dien and Woloszyn, 2005; Tang, 2017), although the protective effect can be weakened by reflective balcony ceilings (Hossam El Dien and Woloszyn, 2004; Wang et al., 2015). The shape and placement of balconies also have to be considered. Hossam El Dien and Woloszyn (2005) found that inclined parapet can provide equivalent reduction in SPL as insulation treatments while multiple rectangular balconies were found to be problematic reflectors (Tang, 2005). Another facade property that has been found to shape the perceived acoustic characteristics of urban spaces is the scattering coefficient of the applied facade materials (Calleri et al., 2018). However, the scattering properties were reported not to have a significant influence on reduction in SPL (Onaga and Rindel, 2002; Badino et al., 2019).

From the literature above, it is obvious that acoustic performance of outdoor urban areas—including inner yards of housing complexes—is affected by architectural design and configuration. Every sound is modified and articulated by the materiality and shape of surrounding surfaces (Maag et al., 2019) and thus, architectural design has a great potential to enhance acoustic comfort in cities (Badino et al., 2019). The challenge is that decisions made regarding the design of an outdoor acoustic space is in the hands of various professionals of the built environment, such as planners, architects, engineers and urbanists, and in some cases, acousticians and sound quality experts. The professionals from various backgrounds have different understandings of the acoustic phenomena and partially different objectives (Brown et al., 2016; Coelho, 2016; Sturm and Bürgin, 2016; Sturm et al., 2019). Often, sound has been seen as an unresolved problem, rather than a planned and designed quality (Maag et al., 2019).

Over the last few decades, consideration of acoustic comfort and soundscape quality within the urban living environment has become more eminent (Schafer, 1993; ISO 12913-1, 2014; Brown et al., 2016; Kang, 2017; ISO 12913-2, 2018), with a focus on the design of a relatively pleasant sound environment instead of focusing on noise emission alone. While the definition of soundscape has been standardized (ISO 12913-1, 2014), the term “acoustic comfort” has a broader and more colorful definition in the literature (Taghipour et al., 2019b). In many studies, the improvement of acoustic comfort has been presented as the general improvement of acoustics, measured in objective acoustical and/or room acoustical parameters (such as lower SPL) (Xiao and Aletta, 2016; Thomas et al., 2018). Other studies have used a subjective evaluation of the acoustic comfort (Yang and Kang, 2005; Kang and Zhang, 2010; Battaglia, 2014; Taghipour et al., 2019b), where acoustic comfort was found to be related to the SPL (Yang and Kang, 2005).

With this background, the present paper investigates whether room acoustical parameters would be proper indicators of acoustic comfort in outdoor areas (i.e., inner yards). Room acoustical parameters were actually developed for performances of music and speech in rooms (ISO 3382-1, 2009; IEC 60268-16, 2011; ODEON, 2018), but have been also used for partially-bounded spaces with open ceilings, such as ancient theaters (ODEON, 2018), historical courtyards for musical performances (Iannace, 2018), and urban spaces (Calleri et al., 2018; Taghipour et al., 2019c). These parameters are not too complex, e.g., a number of them are simple energy ratios, which are available in many simulation software and measurement tools. It is therefore compelling to investigate these parameters for the acoustic quality in partially-bounded outdoor spaces, such as inner yards. This would be particularly useful for architects, acousticians, urban soundscape designers, etc.—who typically have access to simulating software—for the design and development of housing complexes.

The underlying experimental data for the present paper originated from three psychoacoustic laboratory experiments on acoustic comfort in virtual inner yards (Taghipour et al., 2019b). Portions of this study have been published before by Sievers et al. (2018), Taghipour et al. (2019b), and Taghipour et al. (2019c). While Sievers et al. (2018) briefly presented Experiment 1, Taghipour et al. (2019b) reported all three experiments with an analysis of the results with respect to the experimental design variables. Furthermore, Taghipour et al. (2019c) presented a first and brief analysis of the data with respect to the room acoustical parameters, which will now be reported in an expanded length in the present paper. In order to offer the reader a complete picture and to serve as a standalone manuscript, this paper reports the original experiments (Taghipour et al., 2019b), including additional information (e.g., level-time histories and spectral contents of the sound signals, statistical analysis regarding the rating time, etc.), before reporting the analysis with respect to the room acoustical parameters and their association with acoustic comfort.

## 2. Methods

Comfort and discomfort reactions to sounds in virtual (acoustic) outdoor spaces of housing complexes were investigated by means of three psychoacoustic laboratory experiments. The observed “short-term” comfort or discomfort ratings related to acute comfort in response to each stimulus, rather than long-term comfort or well-being which is relevant in *post-hoc* field surveys. Specifically, the term “short-term” refers to the time period during and after an acoustic stimulus' playback and before the next stimulus is presented (Taghipour et al., 2019a,b). Furthermore, the term “acoustic comfort” is subjective (and perceptual) and refers to how comfortable a subject was in the presence of each stimulus in the virtual inner yard.

To investigate possible differences in short-term comfort in inner yards with different building facades, sound propagation was simulated in virtual outdoor spaces. Thereby, single-channel recordings were auralized for a multi-channel playback system (Taghipour et al., 2019b).

Note: This study was approved by Empa's Ethics Committee (Approval Nr. CMI 2018-194).

### 2.1. Experimental Questions

All three experiments presented in this paper investigated which sound sources were associated with short-term acoustic comfort or discomfort. Furthermore and more importantly, the aim of the three experiments was to investigate the effect of the facade's cladding (absorbing vs. reflecting materials) on the perceived acoustic comfort.

- Experiment 1 dealt with the question whether there was a difference between acoustic comfort from sounds in virtual inner yards with reflecting or absorbing facade setups. Furthermore, it was investigated whether receiver (i.e., observer) positions in the yard or on the balcony might be distinctively influenced by the facade covering.
- Experiment 2 investigated the influence of the degree of facade absorption on acoustic comfort. Furthermore, it was investigated whether perceptual differences existed on the balconies of different floors.
- Experiment 3 dealt with the usage of additional facade absorbing materials on the balcony ceilings and their possible influence on the perceived acoustic comfort.

Taghipour et al. (2019b) stated a series of experimental hypotheses resulting from these questions. More details about the design of the experiments and the independent variables in each experiment will be provided in Section 3.

### 2.2. Listening Test Facility

The three experiments presented in this paper were conducted in the listening test facility of Empa, named AuraLab, which has a separate listening and control room allowing for audio-visual supervision to comply with ethical requirements (Taghipour et al., 2019a,b).

**Figure 1:**
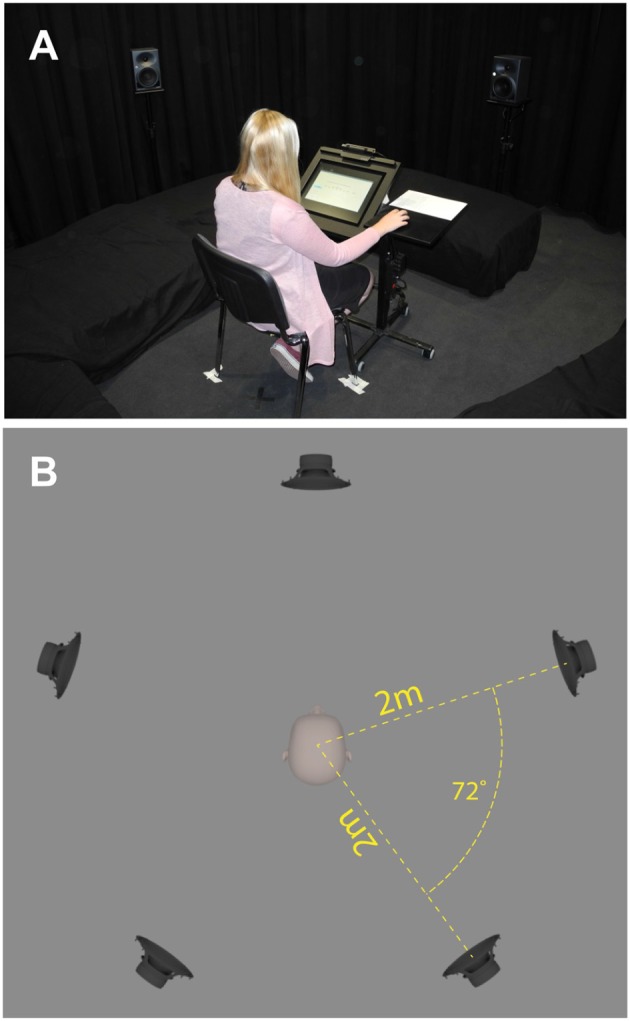
The experimental setup in AuraLab, where the subject was seated in the central listening spot **(A,B)**. For this experiment, additional porous absorbers were put on the floor **(A)**. The stimuli were played back through five satellite loudspeakers and two subwoofers **(B)**.

AuraLab satisfies room acoustical requirements for high-quality audio reproduction in terms of its background noise and reverberation time (Taghipour et al., 2019a). A 3D immersive sound system with 16 separate audio channels is installed in AuraLab. Fifteen loudspeakers “KH 120 A” (Georg Neumann GmbH, Berlin, Germany) are located in a hemispherical arrangement on 3 levels (0, 30, and 60° vertically) in a distance of 2 m from the central listening spot. Bass management is performed by two subwoofers “KH 805” (Georg Neumann GmbH, Berlin, Germany) and a digital signal processor (Taghipour et al., 2019a). Stimuli of the experiments presented here were played back by a 2D setup over the five loudspeakers at the vertical level of 0° (subject's ear level) and both subwoofers (see Figure 1). The reason for this is that ODEON delivers a 2D surround sound—i.e., a five-channel signal—for playback; more details in Section 2.3. Furthermore, the carpeted floor was covered with additional absorbers on the floor (Taghipour et al., 2019b). Figure 1 shows the setup in Auralab, where the subject was seated in the central listening spot.

### 2.3. Recording, Simulation, and Auralization

**Figure 2:**
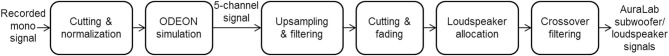
Block diagram of the signal processing steps from recording to playback (Taghipour et al., 2019c).

**Figure 3:**
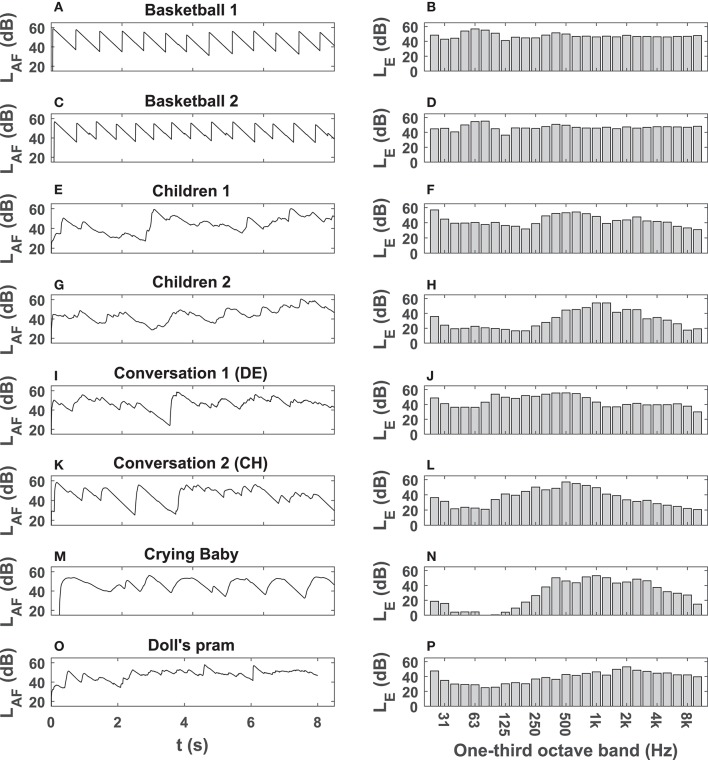
*L*_*AF*_ curves, i.e., A-weighted fast level-time histories (left: **A,C,E,G,I,K,M,O**) and one-third octave spectra (right: **B,D,F,H,J,L,N,P**) of the 8 signals used as ODEON inputs in the three experiments. All signals have an *L*_*Aeq*_ of 50 dB(A).

Figure 2 shows a block diagram of the signal processing from recording to playback. To collect sound sources, single-channel recordings were carried out in a semi-anechoic chamber by means of a B&K 4006 microphone (Brüel & Kjaer, Nærum, Denmark), positioned on the reflecting floor. After suitable 8-s extracts were cut from the recordings, they were normalized to the A-weighted level (i.e., A-weighted equivalent continuous sound level, *L*_Aeq_) of the signal with the largest maximum absolute value of the amplitude (Taghipour et al., 2019b,c). Figure 3 shows level-time histories (*L*_AF_ curves) and one-third octave spectra of the 8-s extracts for a normalized *L*_Aeq_ of 50 dB(A). As shown in Figure 3, several sounds—typical for outdoor living environment—were used in the course of this study. Although, generally, all sounds are neutral in value, they can be determined as pleasant or unpleasant in a particular context and setup by human listeners (ISO 12913-1, 2014; Taghipour et al., 2019b). Prior to the experiments presented in this paper and in a relative approach (i.e., amongst each other), the sounds used in this study were judged by acousticians as to be relatively more or less pleasant. The aim was to facilitate the subjects (of the main experiments) with a variety of sounds that are associated with comfort or discomfort in a laboratory setup.

Room acoustic simulations were done with ODEON v. 14.03 (Odeon A/S, Kgs. Lyngby, Denmark), which uses geometrical acoustics with image-sources and a ray tracing algorithm. Geometrical acoustics methods are currently the most widely used methods in modeling room acoustics, auralization, and outdoor acoustics applications (Georgiou, 2018). Although geometrical acoustics methods have their limitations, e.g., not being able to precisely model wave phenomena such as diffraction (Elorza, 2005), they are popular because of their simplicity and computation efficiency and the ability to model up to high frequencies. Element-based numerical wave-based methods such as Finite Difference Time Domain (FDTD) and Pseudo-Spectral Time Domain (PSTD) in time domain and Finite Element Method (FEM) and Boundary Element Method (BEM) in frequency domain are used for precise acoustic simulations (Hornikx, 2016), but since their solvers need discretized domains and this means a large number of voxels or meshes on a 3D geometry, the computation expense grows much heavily. Hybrid methods have also been used to model higher frequencies using geometrical acoustics and lower frequencies with wave-based methods to obtain a compromise. But still, the most efficient and feasible method seems to be the geometrical acoustics method (Georgiou, 2018), especially with regards to reliable auralization.

An omni-directional sound source was placed in the yard 1.2 m above the ground. Impulse responses were calculated by the software for various observer positions^1^. The simulations were carried out with 200,000 rays and impulse responses of 3.5 s of length. The transition order was set to two. Details about the facade materials are provided by Sievers et al. (2018) and Taghipour et al. (2019b,c). A 2D auralization (2D Surround sound based on first-order B-format Ambisonics) was carried out for five loudspeakers based on the setup in AuraLab (i.e., separated from one another by 72° horizontally, at the vertical angle of 0°). Although ODEON input signals (of different sources) had the same *L*_Aeq_ (see above), the ODEON outputs exhibited diverging *L*_Aeq_, as they possessed unequal spectral and temporal characteristics, to which the virtual rooms reacted differently. The stimuli reported in this paper were simulated considering a single source, one source position, and several observer positions (Taghipour et al., 2019b).

The multi-channel ODEON output signal was upsampled from 44.1 to 48 kHz, as this is a requirement of the playback system. Furthermore, it was low pass (*f*_*c*_ = 10 kHz) and high pass (*f*_*c*_ = 20 Hz) filtered. After being gated with squared-cosine ramps, the multi-channel signal was allocated to the corresponding loudspeakers: front, front-left, front-right, back-left, and back-right. By means of crossover filtering, low-frequency components of the signals were played back over the two subwoofers in the room. Beside the room acoustical simulation in ODEON, all signal processing steps (see Figure 2) were done in the MATLAB environment v. R2016b (MathWorks, Natick, MA, USA).

### 2.4. Reference Inner Yard

**Figure 4:**
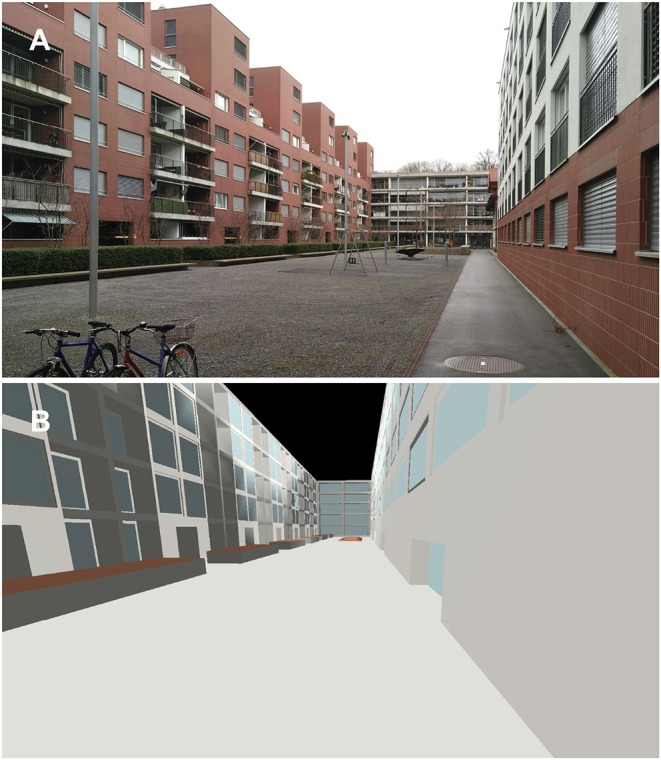
The reference inner yard: **(A)** the building complex and **(B)** its ODEON model.

The reference inner yard used in this study was a simplified 3D model of an existing housing complex in Dübendorf, Switzerland. The geometric model was built in the SketchUp software environment (Trimble Inc., Sunnyvale, CA, USA) and was imported into the ODEON software environment using the plug-in SU2Odeon (Taghipour et al., 2019b). Figure 4 shows the inner yard and its ODEON model. The walls were of brickwork and concrete with large glass windows. Since ODEON works with bounded/closed room models, the inner yard was modeled as an unceiled room (100 × 20 × 20 m), which—for practical reasons—was inserted in a larger box (10 meters away from each side) with a perfect absorbing inner surface, representing free field (Taghipour et al., 2019b).

### 2.5. Experimental Sessions

The three psychoacoustic experiments were conducted as focused listening tests in form of a complete block design with repeated measures. Subjects did the tests individually. After reading the study information, they signed a consent form. Thereafter, they answered the first part of the questionnaire about their hearing and well-being (see Appendix). The subjects were then introduced to the listening test software which guided them through the test. After the listening test, the subjects filled out the rest of the questionnaire (demographic data) (Taghipour et al., 2019b).

Experiment 1 was conducted as a single listening test with 27 subjects (7 females and 20 males, aged between 19 and 57 years old, median 38 years). Experiments 2 and 3 were conducted as two listening tests in one experimental session with 42 subjects (13 females and 29 males, aged between 18 and 64 years old, median 41 years), whereby the order of the Experiments 2 and 3 was (randomly) counterbalanced between the subjects (Taghipour et al., 2019b). It was reported by Taghipour et al. (2019b) that all subjects declared to have normal hearing (self judgment) and to feel well. Since no audiometric test was performed, the subjects were characterized as self-reporting normal-hearing.

### 2.6. Listening Test Software, Procedure, and the Comfort Scale

To familiarize with the sounds and the test software, subjects listened to several orienting and training stimuli. They were chosen such that the subjects were familiarized with the range of different sources, facade types, and sound pressure levels, before starting the main experiment.

- Experiment 1: ten orienting and three training stimuli, out of a total of 60 stimuli.
- Experiment 2: six orienting and four training stimuli, out of a total of 40 stimuli.
- Experiment 3: four orienting and two training stimuli, out of a total of 27 stimuli.

The main listening test began thereafter. For each stimulus, subjects completed the following statement: “In this virtual inner yard and in the presence of this sound, I feel …” (Taghipour et al., 2019b). Their short-term acoustic comfort were recorded during or after stimulus playback on a verbal bipolar 7-point scale: very uncomfortable (−3), uncomfortable (−2), to some extent uncomfortable (−1), neither comfortable nor uncomfortable (0), to some extent comfortable (+1), comfortable (+2), and very comfortable (+3).

To support the neutral category “neither uncomfortable nor comfortable” in its actual purpose as the scale middle category and to avoid its misuse as an avoiding or diverting answer, an additional “don't know” push button was provided to the subjects (Taghipour et al., 2019b). This option was, however, rarely used by the subjects.

The stimuli were played back in a random order after one another, with a 1.2-s break between stimuli after complete playback. By means of a push button, an option was given to the subjects to listen to each stimulus (only) one more time^2^, if they wished to. Subjects rarely made use of this option (Taghipour et al., 2019b).

### 2.7. Statistical Analysis

The statistical analysis was carried out with IBM SPSS Statistics, v. 25 (IBM Corporation, Armonk, USA). Tested effects of the independent variables (and their interactions) on the dependent variable “short-term acoustic comfort” were considered significant if the probability, *p*, of the observed results under the null hypothesis (H0) was less than 0.05 (Taghipour et al., 2019b).

The individual and combined associations of the independent variables (i.e., experimental design variables) on short-term acoustic comfort were investigated as follows (Taghipour et al., 2019b).

- The complete block design of the experiments enabled carrying out repeated-measures multi-factorial analysis of variance (ANOVA) to compare the mean acoustic comfort ratings for different categories of the categorical independent variables. If necessary, failed assumption of sphericity was corrected by the Greenhouse-Geisser method.
- In order to investigate further the directions of the effects, *post-hoc* pairwise comparisons were done by Fisher's protected least significant difference (LSD) test, corrected by the Bonferroni method.
- Furthermore, when helpful, linear mixed-effects models were fitted to the observed data with independent variables of different types; i.e., categorical variables, covariates, and random intercept (comparison of the models by means of Akaike information criterion (AIC) (Akaike, 1998) and Bayesian information criterion (BIC) (Schwarz, 1978); i.e., choosing the model with the lowest AIC/BIC).

Furthermore, it was investigated whether room acoustical parameters and *L*_Aeq_ are good predictors of acoustic comfort. To this aim, the following analyses were carried out.

- Scatter plots (of acoustic comfort as a function of the individual room acoustical parameters) were visually examined.
- Correlations between acoustic comfort and the individual room acoustical parameters were calculated, reporting Pearson's correlation coefficient, *r*, and its significance (Taghipour et al., 2019c). Furthermore, correlations were investigated between mean acoustic comfort and the individual room acoustical parameters.
- Linear mixed-effects models were fitted to the observed data to further investigate the combined analysis of sound source, the individual room acoustical parameters (or *L*_Aeq_), and random intercept as predictors of acoustic comfort. Sound source was taken into this analysis, because this variable is independent of the room and absorption characteristics.
- Furthermore, the data from all three experiments were put together in order to make a combined overall analysis of the results possible.

## 3. Experiments

### 3.1. Experiment 1

Three design variables were used in Experiment 1: inner yard (4 levels), source type (5 levels), and observer position (3 levels) (Sievers et al., 2018). Four yards were modeled in ODEON: the (reflecting) reference yard and three further yards with “exaggerated reflecting building facades,” with “absorbing facades,” and with “exaggerated absorption.” Five different sound sources were tested: a bouncing basketball, a doll's pram, a German conversation, and two sounds of happily playing and laughing children. Three observer points were chosen: two observer points in the yard (1.2 m above the ground, representing the position of someone sitting on a bench), 5 and 20 m away from the source, and one observer point on the second floor balcony about 28 m away from the source. Note that, on average, *L*_Aeq,Balcony_ < *L*_Aeq,20m_ < *L*_Aeq,5m_ and that the observer position at the balcony was considerably more affected by echos and flutter echos (Taghipour et al., 2019b,c). In total, 60 stimuli were prepared for this experiment: 4 × 5 × 3 = 60. The A-weighted equivalent continuous sound levels, *L*_Aeq_, of the auralized stimuli were between 42 and 59 dB(A) [mean *L*_Aeq_ = 52 dB(A)]. Each stimulus was 9 s long.

### 3.2. Experiment 2

Three design variables were used in Experiment 2: the weighted absorption coefficient α_*w*_ (ISO 11654, 1997) (5 levels), source type (4 levels), and observer location (2 levels). α_*w*_ was varied with an approximately exponential progression. To avoid major frequency-dependent differences in absorption properties of materials, a simple material model was chosen, for which the frequency dependency of α remained approximately constant as α_*w*_ was increased (Taghipour et al., 2019b,c). Doing so, the facade was covered with absorbing materials exhibiting α_*w*_ values of 0.05, 0.15, 0.30, 0.55, and 0.95. Four different sources were used: a bouncing basketball, a crying baby, a Swiss German conversation, and a sound of playing children. Two observer points were chosen at the ground floor balcony (patio) and the second floor balcony, 12 and 15 m away from the source. The second floor balcony exhibited lower *L*_Aeq_ than the ground floor balcony (Taghipour et al., 2019b). In total, 40 stimuli were prepared for Experiment 2: 4 × 5 × 2 = 40. The *L*_Aeq_ of the auralized stimuli was between 49 to 64 dB(A) [mean *L*_Aeq_ = 60 dB(A)]. Each stimulus was 10 s long.

### 3.3. Experiment 3

Three design variables were used in Experiment 3: facade α_*w*_ (3 levels), source type (3 levels), and balcony ceiling α_*w*_ (3 levels). α_*w*_ was varied for Experiment 3 between 0.05, 0.30, and 0.95 for the absorption of the facade, as well as the balcony ceiling. Three sound sources were used: a bouncing basketball, a German conversation, and a sound of playing children. The observer was placed at the second floor balcony, 15 m away from the source (Taghipour et al., 2019b,c). In total, 27 stimuli were prepared for Experiment 3: 3 × 3 × 3 = 27. The *L*_Aeq_ of the auralized stimuli was between 49 to 59 dB(A) (mean *L*_Aeq_ = 56 dB(A)). Each stimulus was 10 s long.

## 4. Room Acoustical Parameters

Room acoustical parameters were originally developed to measure and estimate performances of music and speech in rooms (ISO 3382-1, 2009; IEC 60268-16, 2011; ODEON, 2018). However, they are also used in the case of partially-bounded spaces, i.e., spaces with solid floor and walls, but with open ceilings, e.g., from ancient theaters to modern stadiums (Calleri et al., 2018; Iannace, 2018; ODEON, 2018). Since inner yards of building complexes have a similar partially-bounded shape, several classic room acoustical parameters are considered in this paper regarding their ability to predict acoustic comfort. The hypothesis was that they could be used as measures of the quality of the room acoustical experience in the presence of every day life sounds—such as conversations—in outdoor living environments, e.g., inner yards or street canyons. The room acoustical parameters used in this paper will be introduced in the following.

- Speech transmission index, STI, is a quantitative expression of the extent of speech intelligibility (Houtgast and Steeneken, 1973; IEC 60268-16, 2011). STI is derived for an average gender-independent voice spectrum and is expressed as decimal numbers from 0.00 to 1.00. Values in the ranges 0.00–0.30, 0.30–0.45, 0.45–0.60, 0.60–0.75, and 0.75–1.00 correspond to bad, poor, fair, good, and excellent speech intelligibility, respectively.
- Definition (*Deutlichkeit*), D50, is the ratio of the useful energy (the first 50 ms) to the total energy (ISO 3382-1, 2009). It is expressed as percentage values in this paper.
- Clarity (Speech), C50, is the energy ratio before and after 50 ms, expressed in dB, and is stated by ISO 3382-1 (2009) to be appropriate for clarity of speech. Differences between D50 and C50 are that C50 is expressed in dB while D50 is a fraction or percentage and that the integration times for late reverberation are different. C50 is defined as ten times the logarithm of the ratio of the useful energy (of the first 50 ms) to the late energy (after 50 ms).
- Clarity (Music), C80, is an extension of D50 and C50, but is often used for evaluating the space for music (ISO 3382-1, 2009). It is sometimes referred to as “clarity for music.” The only difference between C50 and C80 is the 50-ms or 80-ms limit used in their calculation.
- Early Decay Time, EDT, is a measure that indicates how listeners perceive the reverberation of speech or music at specific listening positions (ISO 3382-1, 2009). It is defined as six times the time during which sound level is attenuated by 10 dB, after turning off the sound source (ISO 3382-1, 2009).
- Lateral Energy Fraction, LF80, is an indication of lateral (bi-directional) energy compared to the early energy (the first 80 ms) (ISO 3382-1, 2009). Late reflections that arrive from lateral directions contribute to the perception of spaciousness (Griesinger, 1997; ISO 3382-1, 2009; ODEON, 2018). These reflections lie between 5 ms and 80 ms. LF80 is an indication of the apparent source width (ASW).
- Dietsch's echo criterion predicts if there is a certain peak in the impulse response that indicates an unwanted audible echo (Dietsch and Kraak, 1986; Kuttruff, 2017).

Room acoustical parameters were calculated in the ODEON environment v. 15 (Odeon A/S, Kgs. Lyngby, Denmark). For D50, C50, C80, and EDT, the average of the values for the two octave bands centered at 500 and 1,000 Hz was used here, as recommended by ISO 3382-1 (2009). A similar approach was applied to EchoD. Furthermore, average LF80 values were used which were provided by the ODEON (ODEON, 2018), according to recommendations by ISO 3382-1 (2009). The LF80 average values were calcultaed over 125, 250, 500, and 1,000 Hz octave bands (ISO 3382-1, 2009; ODEON, 2018).

## 5. Results

This section briefly discusses the most important results delivered by Taghipour et al. (2019b) accompanied by further experimental results, before reporting the analysis with the room acoustical parameters.

### 5.1. Independent Design Variables

**Figure 5:**
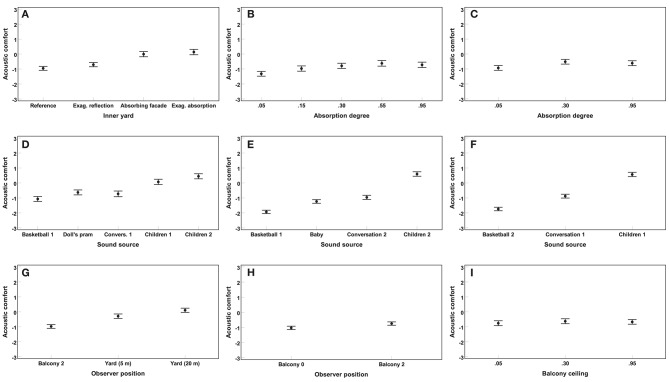
Results of Experiments 1 (left: **A,D,G**), 2 (middle: **B,E,H**), and 3 (right: **C,F,I**): Mean acoustic comfort ratings across subjects and their 95% confidence intervals are shown on the ordinate for different design parameters along the abscissa. In Experiment 1, three variables, i.e., inner yard type (4 levels), sound source (5 levels), and observer position (3 levels), were tested. In Experiment 2, three variables, i.e., facade's weighted absorption coefficient α_*w*_ (5 levels), sound source (4 levels), and observer position (2 levels), were tested. In Experiment 3, three variables, i.e., facade's weighted absorption coefficient α_*w*_ (3 levels), sound source (3 levels), and balcony ceiling's weighted absorption coefficient α_*w*_ (3 levels), were tested (Taghipour et al., 2019b).

Figure 5 illustrates mean acoustic comfort ratings and their 95% confidence intervals for data from Experiments 1, 2, and 3, originally presented by Taghipour et al. (2019b).

#### 5.1.1. Results of Experiment 1

In total, 1620 (i.e., 27 subjects × 60 stimuli) acoustic comfort ratings were collected in Experiment 1. Significant main effects on acoustic comfort were found for all three design variables, i.e., inner yard [*F*_(2.2,57.2)_ = 49.6], sound source [*F*_(3.1,79.3)_ = 33.1], and observer position [*F*_(1.2,31.9)_ = 25.8], all *p* < 0.001 (Taghipour et al., 2019b).

Compared to the two reflecting inner yards, acoustic comfort was rated higher for the two absorbing inner yards, *p* < 0.001. No further differences were found between the inner yards, all *p* > 0.05. Acoustic comfort was rated higher for the two children sounds than for the three other sound sources, all *p* < 0.001. No further significant differences were found between the sound sources, all *p* > 0.05. Furthermore, acoustic comfort ratings revealed to be significantly different for the three observer positions, all *p* < 0.01. The observer positions at 20 m distance in the yard and at the second floor balcony were found to be “more” and “less” comfortable than the position at 5 m distance in the yard, respectively (Taghipour et al., 2019b).

Furthermore, a series of significant interactions were reported and discussed extensively by Taghipour et al. (2019b).

#### 5.1.2. Results of Experiment 2

In total, 1678 (i.e., 42 subjects × 40 stimuli − 2 missing data points) acoustic comfort ratings were collected in Experiment 2. Significant main effects on acoustic comfort were found for all three design variables, i.e., facade's α_*w*_ [*F*_(3.0,121.8)_ = 21.0], sound source [*F*_(2.7,112.1)_ = 71.6], and observer position [*F*_(1.0,41.0)_ = 29.3], all *p* < 0.001 (Taghipour et al., 2019b).

A linear mixed-effect model was fitted to the data of Experiment 2 to investigate the effect of facade's α_*w*_ on acoustic comfort (also considering sound source, observer position, and subjects' random intercept). A parabolic relationship was found. That is, acoustic comfort was rated the highest for moderate (i.e., middle) α_*w*_ values. Basketball 1 and children 2 were rated as least and most comfortable sounds, respectively, all *p* < 0.01. No further significant difference was found with respect to sound sources, *p* > 0.05. Acoustic comfort was rated higher for balcony 2 than for balcony 0, *p* < 0.01 (Taghipour et al., 2019b).

Furthermore, a series of significant interactions were reported and discussed extensively by Taghipour et al. (2019b).

#### 5.1.3. Results of Experiment 3

In total, 1134 (i.e., 42 subjects × 27 stimuli) acoustic comfort ratings were collected in Experiment 3. Significant main effects on acoustic comfort were found for facade's α_*w*_ [*F*_(1.4,56.6)_ = 9.5] and sound source [*F*_(2.0,80.0)_ = 105.7], all *p* < 0.01. Balcony ceiling's α_*w*_ was not found to affect acoustic comfort significantly, although such a non-significant tendency could be observed [*F*_(2.0,80.3)_ = 2.8], *p* = 0.07 (Taghipour et al., 2019b).

Fitted by a linear mixed-effect model, the effect of facade's α_*w*_ on acoustic comfort was very similar to that in Experiment 2. Balcony ceiling's α_*w*_, however, was not found to be significantly contributing to the model. Nevertheless, in the absence of any absorbers on the facade, absorbing balcony ceilings tended to improve acoustic comfort. Regarding sound source, basketball 2 and children 1 were rated to be less and more comfortable than conversation 1, respectively, all *p* < 0.001 (Taghipour et al., 2019b).

### 5.2. Rating Time

**Figure 6:**
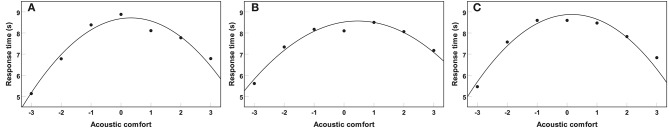
Mean rating time as a function of acoustic comfort rating for **(A)** Experiment 1, **(B)** Experiment 2, and **(C)** Experiment 3.

Figure 6 shows mean rating time (response time) as a function of acoustic comfort. For all three experiments, a parabolic relationship can be observed. That is, when subjects felt very uncomfortable or very comfortable, they gave their response faster than when they rated their comfort in response to the acoustic stimuli in the middle range of the scale. The absolute fastest mean ratings (in all three experiments) were collected when the stimuli was perceived to be very uncomfortable.

### 5.3. Playback Sequence

It was investigated whether playback sequence (i.e., the order of stimuli's playback) affected short-term acoustic comfort. Table 1 shows Pearson's correlation coefficient, *r*, between acoustic comfort and playback sequence.

**Table 1:** Pearson's *r* for correlations between playback sequence and acoustic comfort.

	**Exp. 1**	**Exp. 2**	**Exp. 3**	**Overall**
Sequence	−0.02	−0.03	−0.11^**^	−0.06^**^

All significant cases are significant at the level of

** *p < 0.01*.

**Figure 7:**
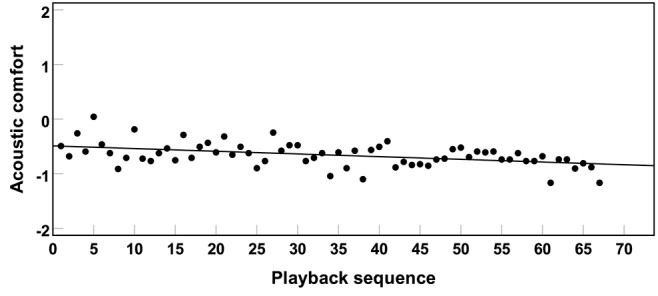
Mean acoustic comfort ratings (for the combined data of the three experiments) across subjects and sound sources as a function of playback sequence. Notice that the second and the third experiments were conducted in one experimental session. Therefore, in the combined analysis, the playback number was registered from 1 to 67 (i.e., 40 and 27 stimuli in Experiments 2 and 3, respectively).

It can be observed in Figure 7 that, overall, acoustic comfort decreased slightly with increasing playback sequence. Nevertheless, as Table 1 shows, correlation between acoustic comfort and playback sequence was either weak or non-significant. The effect of playback sequence on acoustic comfort was not further analyzed in linear mixed-effect models fitted to the observed data.

### 5.4. Room Acoustical Parameters

#### 5.4.1. Scatter Plots and Correlations

**Figure 8:**
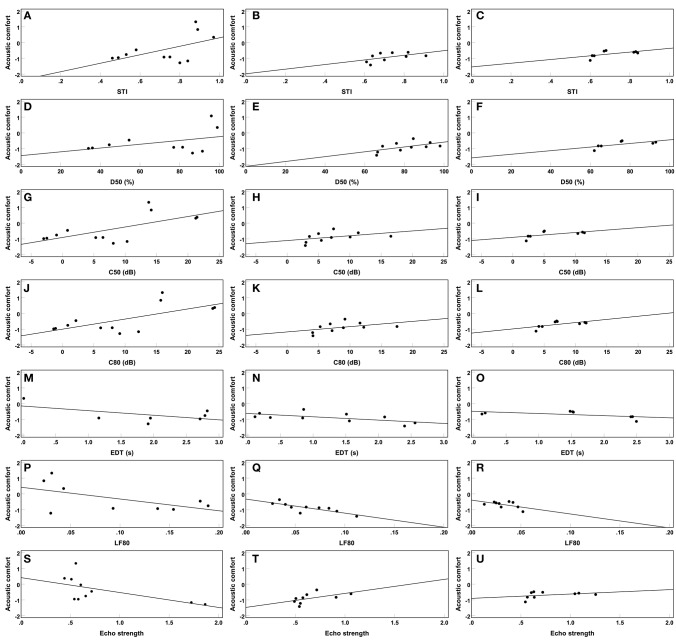
Scatter plots showing mean acoustic comfort ratings across subjects and sound sources as a function of the individual room acoustical parameters. The lines indicate simple linear regressions based on the respective room acoustical parameters. The left panel (i.e., **A,D,G,J,M,P,S**), the middle panel (i.e., **B,E,H,K,N,Q,T**), and the right panel (i.e., **C,F,I,L,O,R,U**) show the results of Experiments 1, 2, and 3, respectively.

Figure 8 shows a series of scatter plots of mean acoustic comfort rating as a function of the individual room acoustical parameters. Except for the echo criterion by Dietsch and Kraak (1986), the slope sign (i.e., positiveness vs. negativeness) for each room acoustical parameter is consistent for all three experiments. This is further quantified by the significant correlations between acoustic comfort and the individual parameters. Pearson's correlation coefficient, *r*, is reported in Table 2 for correlations between acoustic comfort ratings and the room acoustical parameters of Figure 8. Except for the echo criterion by Dietsch and Kraak (1986) in Experiment 3, all correlations were found to be significant. Note that, whereas, for Experiment 1, rather moderate correlations were observed, for Experiments 2 and 3, correlations were very weak. Furthermore, strong correlations were found between mean acoustic comfort and the individual room acoustical parameters (see Table 2).

**Table 2:** Pearson's *r* for correlations between room acoustical parameters and acoustic comfort (and mean acoustic comfort).

		**STI**	**D50**	**C50**	**C80**	**EDT**	**LF80**	**EchoD**
Experiment 1	Acoustic comfort	0.29^**^	0.26^**^	0.32^**^	0.33^**^	−0.32^**^	−0.23^**^	−0.24^**^
	(mean acoustic comfort)	(0.55)	(0.41)	(0.66)	(0.67)	(−0.58)	(−0.56)	(−0.59)
Experiment 2	Acoustic comfort	0.08^**^	0.10^**^	0.08^**^	0.08^**^	−0.11^**^	−0.14^**^	0.09^**^
	(mean acoustic comfort)	(0.53)	(0.57)	(0.43)	(0.46)	(−0.63)	(−0.80)	(0.54)
Experiment 3	Acoustic comfort	0.06^*^	0.08^**^	0.06^*^	0.07^*^	−0.07^*^	−0.07^*^	0.04
	(mean acoustic comfort)	(0.59)	(0.66)	(0.59)	(0.60)	(−0.59)	(−0.53)	(0.35)
Overall	Acoustic comfort	0.18^**^	0.15^**^	0.20^**^	0.21^**^	−0.18^**^	−0.11^**^	−0.08^**^
	(mean acoustic comfort)	(0.51)	(0.38)	(0.59)	(0.62)	(−0.46)	(−0.31)	(−0.30)

* *p < 0.05*.

** *p < 0.01*.

While short-term acoustic comfort increased with increasing STI, D50, C50, and C80, it decreased with increasing EDT and LF80 (see Figure 8 and Table 2). Since the correlation between acoustic comfort and the EchoD is rather inconsistent for the results of the three experiments reported here—i.e., negative correlation in Experiment 1, positive correlation in Experiment 2, and no significant correlation in Experiment 3—(see Figure 8 and Table 2), this criterion was considered not to be a proper predictor of acoustic comfort. Therefore, the echo criterion by Dietsch and Kraak (1986) was not investigated in the further analysis.

#### 5.4.2. Linear Mixed-Effect Models Including Individual Room Acoustical Parameters

Several linear mixed-effect models were fitted to the observed data to investigate the relationship between the dependent variable acoustic comfort and the individual room acoustical parameters. That is, instead of the physical design parameters (i.e., inner yard, observer position, and α_*w*_ of the facade and the balcony ceiling), individual room acoustical parameters were considered in the models as independent variables, accompanied by the categorical independent variable sound source and subjects' random intercept (Taghipour et al., 2019c). Note that, since a majority of the correlations between individual room acoustical parameters and acoustic comfort in Table 2 are weak, it would be careless to interpret the room acoustical parameters as good predictors of acoustic comfort without considering other independent variables (such as sound source). That is, only if, in the presence of sound source and subject's random intercept, the room acoustical parameters were significant predictors in the fitted linear mixed-effect models, their prediction ability should be taken seriously.

A series of models were fitted to the data which considered sound source, random intercept, and one room acoustical parameter. For the results of all three experiments, including STI, D50, C50, C80, EDT, or LF80 in the models contributed significantly to predict the corresponding acoustic comfort ratings. The direction of their effect was analogous to correlations in Table 2. That is, if they were correlated with acoustic comfort positively (or negatively), the corresponding β in the linear mixed-effect model was positive (or negative); see Table 3. The linear mixed-effect models were defined by the following equations:

(1) $$\begin{array}{l} y_{ik}=\mu +\tau_{\text{Src},i}+\beta \cdot RAP+u_{k}+\epsilon_{ik}. \end{array}$$

In Equation (1), *y*_*ik*_ is the dependent variable acoustic comfort, μ is the overall grand mean, τ_Src,*i*_ denotes the categorical variable source type (five levels in Experiment 1: *i* = 1 − 5, four levels in Experiment 2: *i* = 1 − 4, three levels in Experiment 3: *i* = 1 − 3), *RAP* is the continuous variable (each individual) room acoustical parameter, and β is its regression coefficient. The (unstructured) random effect term *u*_*k*_ is subjects' random intercept (Experiment 1: *k* = 1 − 27, Experiment 2: *k* = 1 − 42, Experiment 3: *k* = 1 − 42). Finally, the error term ϵ_*ik*_ is the random deviation between observed and expected values of *y*_*ik*_. All parameters contributed significantly to most of the models (all *p* < 0.05). Only for Experiment 1 and in the case of EDT and LF80, the random intercept (i.e., subject) was a non-significant predictor. That is, only in these two cases (out of a total of 18 cases), the significant linear mixed-effect model equations did not include *u*_*k*_. All models were better than the basic model with sound source and random intercept (without room acoustical parameters). That is, adding individual room acoustical parameters improved the basic models significantly and led to lower AIC and BIC (Schwarz, 1978; Akaike, 1998).

**Table 3:** Values of β in Equation (1) for different room acoustical parameters in the three experiments (all *p* < 0.01).

	**STI**	**D50**	**C50**	**C80**	**EDT**	**LF80**
Exp. 1	2.76	1.82	0.07	0.06	−0.44	−6.16
Exp. 2	1.50	1.54	0.03	0.03	−0.21	−8.97
Exp. 3	1.10	1.04	0.03	0.04	−0.12	−8.80
Overall	2.27	1.65	0.06	0.06	−0.31	−6.73

Table 3 shows the values of β in Equation 1 for each room acoustical parameter and experiment. Furthermore, β values are listed for the combined (i.e., overall) analysis of all three experiments; see Section 5.4.3. Note that, in total, there are 24 models in the form of Equation 1 and their complete reporting would not be possible in this paper.

It is important to compare the β values from linear-mixed effect models reported in Table 3 with the simple linear regressions shown in Figure 8. While the linear mixed-effect models additionally consider the strong effect of sound source and random subject intercept, the relationships between acoustic comfort and individual room acoustical parameters resemble those reported in Figure 8. The signs of the relationships (i.e., positive or negative correlations) are identical in Table 3 and Figure 8. Furthermore, for each individual room acoustical parameter, the differences between the regression coefficients (i.e., the slopes) in the three experiments show a similar pattern. That is, even considering other predictors, a fairly similar relationship holds between acoustic comfort and the individual room acoustical parameters as to that from Figure 8.

#### 5.4.3. Linear Mixed-Effect Models for the Combined Data of the Three Experiments

As mentioned above, linear mixed-effect models were established with the “combined data” from the three experiments (e.g., see Tables 2, 3, “overall”). The same Equation (1) was found to be appropriate for all individual room acoustical parameters; see Table 3 for β values. In this case as well, all models were better than the basic counterpart with sound source and random intercept (without room acoustical parameters). Based on AIC and BIC, the strongest to the weakest models were with C80, C50, EDT, STI, LF80, and D50. A similar model with *L*_Aeq_ instead of the individual room acoustical parameters was found to be weaker than all the other models and than the basic model.

### 5.5. Multiple Room Acoustical Parameters

Since room acoustical parameters are (partially) strongly and significantly correlated with each other (e.g., in many cases: Pearson's *r* > 0.90, *p* < 0.01), special care is needed when multiple room acoustical parameters are being considered in a model. Possible collinearities must be avoided. A series of models were fitted to the observed data considering sound source, random intercept, and multiple room acoustical parameters, with or without *L*_Aeq_. Several models were found significant. While these models will not be introduced here in further details, it should be noted that adding more than two room acoustical parameters simultaneously typically did not improve the models any further.

### 5.6. *L*_Aeq_

Generally, short-term acoustic comfort decreased with increasing *L*_Aeq_. This confirms findings reported by Yang and Kang (2005). However, the picture was more complicated than this statement.

In the course of the further analyses, for each experiment, a model was fitted to the data considering sound source, random intercept, and *L*_Aeq_. For Experiment 1, *L*_Aeq_ contributed significantly in the model, however, only in interaction with sound source. The following model was found to be appropriate for Experiment 1:

(2) $$\begin{array}{l} y_{ik}=\mu +\tau_{\text{Src},i}+\beta \cdot L_{\text{Aeq}}+\beta_{\text{Src},i}\cdot L_{\text{Aeq}}+\epsilon_{ik}. \end{array}$$

In Equation (2), *L*_Aeq_ is the continuous variable *L*_Aeq_ and β is its regression coefficients. Subjects' random intercept was not found to be significantly contributing to the model. Model coefficients are shown in Table 4.

**Table 4:** Experiment 1: model coefficients (Coeff.), their 95% CI, and probabilities (*p*) of the linear mixed-effects model for acoustic comfort.

**Parameter**	**Symbol**	**Coeff**.	**95% CI**	***p***
Intercept	μ	0.400	[−4.018;4.818]	0.859
Sound source	τ_Src,*i*_ = Basketball 1	1.546	[−1.667;4.758]	0.345
	τ_Src,*i*_ = Doll's pram	3.235	[0.608;5.86]	0.016
	τ_Src,*j*_ = Conversation 1	−3.216	[−5.771;−0.661]	0.014
	τ_Src,*j*_ = Children 1	−2.747	[−5.245;−0.250]	0.031
	τ_Src,*j*_ = Children 2	0^a^		
*L*_Aeq_	β	0.001	[−0.034;0.035]	0.961
Source × *L*_Aeq_	β_Src,*i*_ = Basketball 1	−0.068	[−0.136;0.001]	0.051
	β_Src,*i*_ = Doll's pram	−0.090	[−0.143;−0.038]	0.001
	β_Src,*i*_ = Conversation 1	0.041	[−0.009;0.091]	0.107
	β_Src,*i*_ = Children 1	0.047	[−0.002;0.095]	0.058
	β_Src,*i*_ = Children 2	0^a^		

*The parameters and symbols are explained in Equation (2)*.

a *Redundant coefficients are set to zero*.

For Experiments 2 and 3, *L*_Aeq_ contributed significantly in the model, however, only without interaction with sound source. The following model was found to be appropriate for these two experiments:

(3) $$\begin{array}{l} y_{ik}=\mu +\tau_{\text{Src},i}+\beta \cdot L_{\text{Aeq}}+u_{k}+\epsilon_{ik}. \end{array}$$

Model coefficients for the analysis of Experiments 2 and 3 corresponding to Equation (3) are shown in Tables 5, 6.

**Table 5:** Experiment 2: model coefficients (Coeff.), their 95% CI, and probabilities (*p*) of the linear mixed-effects model for acoustic comfort.

**Parameter**	**Symbol**	**Coeff**.	**95% CI**	***p***
Intercept	μ	10.709	[8.513;12.904]	0.000
Sound source	τ_Src,*i*_ = Basketball 1	−4.197	[−4.586;−3.809]	0.000
	τ_Src,*i*_ = Baby	−2.100	[−2.260;−1.941]	0.000
	τ_Src,*j*_ = Conversation 2	−1.894	[−2.058;−1.729]	0.000
	τ_Src,*j*_ = Children 2	0^a^		
*L*_Aeq_	β	−0.164	[−0.199;−0.129]	0.000

*The parameters and symbols are explained in Equation (3)*.

a *Redundant coefficients are set to zero*.

**Table 6:** Experiment 3: model coefficients (Coeff.), their 95% CI, and probabilities (*p*) of the linear mixed-effects model for acoustic comfort.

**Parameter**	**Symbol**	**Coeff**.	**95% CI**	***p***
Intercept	μ	6.646	[3.468;9.824]	0.000
Sound source	τ_Src,*i*_ = Basketball 2	−2.925	[−3.280;−2.570]	0.000
	τ_Src,*j*_ = Conversation 1	−1.524	[−1.678;−1.369]	0.000
	τ_Src,*j*_ = Children 1	0^a^		
*L*_Aeq_	β	−0.107	[−0.162;−0.051]	0.000

*The parameters and symbols are explained in Equation (3)*.

a *Redundant coefficients are set to zero*.

While for the latter two experiments, for each sound source, acoustic comfort decreased with increasing *L*_Aeq_, for Experiment 1, the picture was more complex (Taghipour et al., 2019c). Relatively unpleasant sound sources showed a similar pattern with increasing *L*_Aeq_ as for Experiments 2 and 3. However, acoustic comfort was slightly increased with increasing *L*_Aeq_ for the two pleasant children sounds. Taghipour et al. (2019b) offered a detailed discussion of the effect of *L*_Aeq_ on acoustic comfort and the implications of the differences between the mean *L*_Aeq_ of the three experiments.

## 6. Discussion

### 6.1. Discussion and Implications of the Results

It was mentioned in Section 1 that, in the literature, the term “acoustic comfort” has been used with various definitions and been measured with different subjective and objective methods. Taghipour et al. (2019b) offered a discussion on the differences in approaches and measures related to this term. The short-term acoustic comfort here was rated subjectively on a bipolar 7-point scale (see Section 2.6). Compared to any other methods and definitions in the literature, this would be more comparable to the acoustic comfort used by Yang and Kang (2005) which was rated on a bipolar 5-point scale.

Moderate facade absorption was found to increase acoustic comfort. Both reflective and too absorptive facades were associated with low acoustic comfort ratings. While in Experiments 2 and 3, the absorption degree of the whole facade (beside glass windows) was varied systematically (i.e., based on α_*w*_), in Experiment 1, different surfaces were either reflective or absorptive. This suggests that, in the design stage, both approaches could be useful: applying materials with moderate absorption characteristics (here middle-ranged α_*w*_ values) for the facade or using highly absorbing materials for only a selected portion of the facade. Furthermore, in the absence of any facade absorption, absorbing materials on the balcony ceilings tended to increase acoustic comfort on the balconies. That would be a simple and cheap solution which can also be applied post construction (Taghipour et al., 2019b). The results of this study seem to be generally in accord with findings in other studies which suggested use of absorbing materials on the facade and/or balconies (Lee et al., 2007; Hornikx and Forssén, 2009; Yeung, 2016; Calleri et al., 2017; Badino et al., 2019).

A dominant factor that influenced the acoustic comfort in the virtual inner yards was the sound source, i.e., the content of the sound present in the yard. While almost all sounds yielded a negative rating concerning the perceived acoustic comfort, relatively pleasant and relatively unpleasant sounds were found to increase and decrease acoustic comfort, respectively. Enabling facilities that invite relatively pleasant sounds, e.g., playing children as well as water features, birds and vegetation (Jeon et al., 2010; De Coensel et al., 2011; Taghipour and Pelizzari, 2019) and avoiding facilities which encourage relatively unpleasant sounds and noisy activities (such as basketball) might improve the overall acoustic comfort in inner yards. This point should be, however, treated with caution, due to inherent differences between short-term responses in a laboratory setup and long-term effects of the sounds in a living environment (Schäffer et al., 2016; Taghipour et al., 2019a).

A number of classic room acoustical parameters were found to be significant predictors of short-term acoustic comfort in linear-mixed effect models fitted to the observed data, including sound source, individual room acoustical parameters, and subject's random intercept. Only the echo criterion proposed by Dietsch and Kraak (1986) was not found to be a significant predictor of acoustic comfort. The room acoustical parameters investigated here are an initial set of acoustic indicators, however, not originally defined for acoustic scenarios in outdoor areas. The main purpose of the analysis in this paper was whether they could serve as indicators of acoustic performance in yards. The results support this hypothesis. In linear mixed-effect models with multiple room acoustical parameters, no more than two room acoustical parameters were found to be needed simultaneously. On the one hand, this suggests that the list presented here could be shortened and optimized. Hereby the results suggest that C50 (or C80), EDT, and STI would be the most important room acoustical parameters related to acoustic comfort. On the other hand, there might be other acoustic indicators which operate similarly or more successfully for this purpose. It might also be possible to define new parameters based on the results here and from similar future studies.

It was reported in Section 4 that—for the majority of room acoustical parameters used here—the statistical analysis was done with averaged room acoustical parameter data. That is, the average for the two octave bands centered at 500 and 1,000 Hz was chosen as representative for each room acoustical parameter, as recommended by ISO 3382-1 (2009). This suggestion, however, holds for performance spaces, not for outdoor living environments. In order to test whether this averaging was suitable in the case of the present study, an alternative averaging system has also been tested, whereby the average for the four octave bands centered at 250, 500, 1,000, and 2,000 Hz was used. The results of all statistical analyses were, however, stronger with the 500–1,000 averaged data. Therefore, only these analyses were reported in this paper.

Another set of established acoustic indicators are the so called psychoacoustic parameters. Rather than being based only on the objective physical characteristics of the acoustic situation, they are derived from subjective perception of sound by humans. The authors suggest to also investigate exploiting psychoacoustic parameters in future investigations to model acoustic comfort.

Acoustic comfort decreased slightly with increasing playback sequence (see Section 5.3). This might be because the sound exposure level (*L*_AE_) increased with increasing playback sequence. That is, subjects were gradually exposed to a higher number of sounds, which increased the cumulative *L*_AE_. It is reasonable to assume that higher (or cumulative) sound pressure levels are generally associated with lower acoustic comfort ratings (see Section 5.6 for a discussion on the effect of sound pressure level on acoustic comfort). This is consistent with other laboratory experiments, whereby “short-term noise annoyance” was reported either not to be significantly affected by increasing playback sequence or to increase with it (Schäffer et al., 2016, 2019; Taghipour and Pelizzari, 2019; Taghipour et al., 2019a). Hereby, it is noted that increased noise annoyance is typically associated with decreased acoustic comfort (Yang and Kang, 2005). For laboratory psychoacoustic experiments, the effect of the playback sequence indicates that a randomization of the playback list for the subjects—and counterbalancing where needed—is necessary, as carried out in the course of this study.

### 6.2. Limitations

The room acoustical simulation method used by ODEON is based on a geometrical approach with image sources and ray tracing. This method can have limitations, for example, regarding diffraction. Furthermore, while AuraLab is capable of a 3D playback over up to 15 loudspeakers, the 2D Surround sound ODEON output is limited to a horizontal plane at the ears' level. It should, however, be possible to use the B-Format ODEON output and decode the four-channel B-Format signal as a 3D scenario. This was not done in this study.

The experiments presented here did not include any visual stimuli. This made an investigation of the acoustical perception possible without any confounding effects of additional visual stimuli. Nevertheless, there is evidence for aural-visual interactions which might influence the overall perception (including comfort) in the laboratory (Viollon et al., 2002; Maffei et al., 2013a,b; Ernst and Bülthoff, 2014; Schäffer et al., 2019).

While interpreting the outcome of this study, one should consider the general differences between laboratory experiments and on-site experience and that most of the subjects of the three experiments work for authors' research institute. It should be further differentiated between short-term and long-term comfort.

The current study was carried out with single static sources in each stimuli. However, typically a mixture of (static and moving) sounds in background and foreground are present in reality. This limitation occurred partially because of computational limitations in ODEON and partially in order to reduce complexity. This should be improved in future studies.

The statistical models presented in this paper were fitted on the observed data. They investigated the relationship between the dependent variable acoustic comfort and a series of independent variables or alternatively the mediating variables room acoustical parameters. Therefore, the conclusions of this paper should not be generalized. In order to have a generic predictive model linking the room acoustical parameters to the acoustic comfort a larger amount of data would be needed and the predictive models would need to be further validated.

## 7. Conclusions

This paper investigated to what extent classic room acoustical parameters are suitable to predict perceived acoustic comfort in outdoor spaces of housing complexes. Subjective acoustic comfort ratings were collected in the course of three psychoacoustic experiments in the laboratory. The acoustic stimuli consisted of sounds from virtual inner yards of housing complexes. The analysis revealed that, beside the strong effect of the sound source (i.e., relative pleasantness or unpleasantness of the sound source), also *L*_Aeq_ and a series of room acoustical parameters could be used as predictors/indicators of acoustic comfort. In the design stage of housing complex projects, the estimated values for relevant room acoustical parameters could indicate the degree of subjective acoustic comfort. Thus, design changes which lead to an optimization (maximization/minimization) of estimated room acoustical parameters could be useful in improving acoustic performance. This should be helpful for architects, urban soundscape designers, and acousticians to improve the perceived acoustic comfort for the residents of housing complexes.

## Data Availability Statement

The datasets generated for this study will not be made publicly available. Requests to access these datasets should be directed to the corresponding author.

## Ethics Statement

The studies involving human participants were reviewed and approved by Empa's Ethics Committee (Approval Nr. CMI 2018-194). The participants provided their written informed consent to participate in this study.

## Author Contributions

This project was conceptualized and supervised by KE and AT and managed by AT. Experiments were designed by AT, KE, and TS and were carried out by TS and AT. Data analysis was done by AT and graphs were prepared by AT and TS. The analysis with room acoustical parameters was carried out by AT and SA. A first draft of this paper was conceptualized and written by AT, AG, and SA. All authors participated in reviewing the paper.

### Conflict of Interest

The authors declare that the research was conducted in the absence of any commercial or financial relationships that could be construed as a potential conflict of interest.

## Footnotes

^1^The observer positions in the yard, 1.2 m above the ground, represented a person sitting on a bench for the purpose of recreation/relaxing. As—from an experimental design perspective—the authors had decided to have one source position for all the sound sources, they decided this height to represent playing children, as well as conversations, etc., therefore, 1.2 m above the ground.

^2^The repetition was offered “only one more time” in order to prevent a too lengthy experiment. Giving subjects the opportunity to listen to the stimuli “as many times as they wish to” would cause two problems. First, the experimental schedule would not be totally under experimenters' control. That is, there would be a need for planning very long sessions, just to avoid any overlaps. This would not be an efficient use of the laboratory setup and staff. Second, a very essential point in laboratory experiments is to provide all subjects with a similar and controlled situation/setup in the lab. This essential point would not be satisfied, if one subject rarely used any repetitions and another subject regularly used several repetitions. They would not be participating in the same listening test scheme.

## Appendix

The first part of the questionnaire included six questions about the subjects' hearing (4 questions) and well-being (2 questions). The answers to these questions were used as inclusion/exclusion criteria. The data of no subject had to be excluded from the final analysis. The questions were:

- How good is your hearing? (5-point bipolar scale)
- Do you suffer from any hearing loss, hearing illnesses or ear noises (e.g., tinnitus)? (Y/N)
- Do you use a hearing aid? (Y/N)
- Do you have a cold at the moment? (Y/N)
- Are you feeling healthy and well? (Y/N)
- Are you feeling very tired? (Y/N)
